# Quantitative Analysis of Abamectin, Albendazole, Levamisole HCl and Closantel in Q-DRENCH Oral Suspension Using a Stability-Indicating HPLC-DAD Method

**DOI:** 10.3390/molecules27030764

**Published:** 2022-01-24

**Authors:** Hazim M. Ali, Mohammed Gamal, Mohammed M. Ghoneim, Lobna Mohammed Abd Elhalim

**Affiliations:** 1 Department of Chemistry, College of Science, Jouf University, P.O. Box 2014, Sakaka 72388, Aljouf, Saudi Arabia; hmali@ju.edu.sa; 2 Pharmaceutical Analytical Chemistry Department, Faculty of Pharmacy, Beni-Suef University, Alshaheed Shehata Ahmed Hegazy St., Beni-Suef 62574, Egypt; 3 Department of Pharmacy Practice, College of Pharmacy, AlMaarefa University, Ad Diriyah 13713, Riyadh, Saudi Arabia; mghoneim@mcst.edu.sa; 4 Analytical Chemistry Department, Central Administration of Drug Control, Egyptian Drug Authority, 51 Wezaret Al Zeraa Street, Agouza, Giza 12311, Egypt; lobna_nodcar@yahoo.com

**Keywords:** Abamectin, Albendazole, Levamisole HCl, Closantel, anti-parasitic drugs, stability study, HPLC, Q-DRENCH suspension

## Abstract

Combination therapy of many anthelmintic drugs has been used to achieve fast animal curing. Q-DRENCH is an oral suspension, containing four different active drugs against GIT worms in sheep, commonly used in Australia and New Zeeland. The anti-parasitic drugs are Albendazole (ALB), Levamisole HCl (LEV), Abamectin (ABA), and Closantel (CLO). The main purpose of this study is to present a new simultaneous stability-indicting HPLC-DAD method for the analysis of the four drugs. The recommended liquid system was 1 mL of Triethylamine/L water, adjusting the pH to 3.5 by glacial acetic acid: acetonitrile solvent (20:80, *v*/*v*). Isocratic elusion achieved the desired results of separation at a 2 mL/min flow rate using Zorbax C-18 as a stationary phase. Detection was performed at 210 nm. The linearity ranges were 15.15 to 93.75 μg/mL for ALB, 25 to 150 μg/mL for LEV, 30 to 150 μg/mL for ABA, and 11.7 to 140.63 μg/mL for CLO. Moreover, the final greenness score was 0.62 using the AGREE tool, which reflects the eco-friendly nature. Moreover, the four drugs were determined successfully in the presence of their stressful degradation products. This work presents the first chromatographic method for simultaneous analysis for Q-DRENCH oral suspension drugs in the presence of their stressful degradation products.

## 1. Introduction

**Figure 1:**
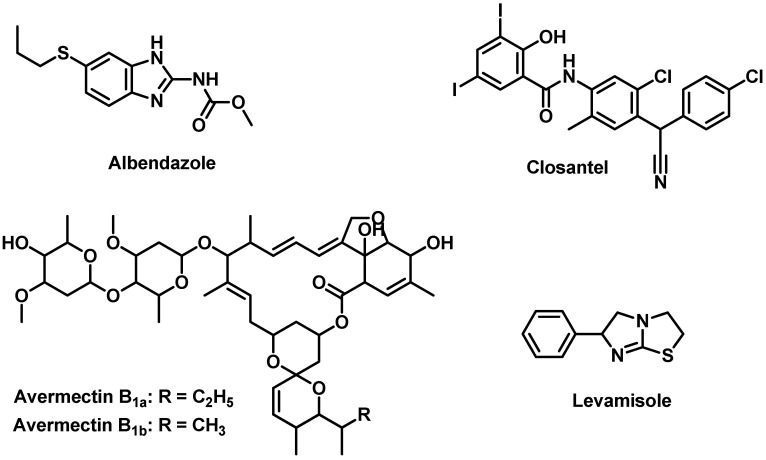
Chemical structures of Albendazole (ALB), Levamisole HCl (LEV), Abamectin (ABA), and Closantel (CLO).

Gastrointestinal infection with nematodes is one of the main causes of financial losses in sheep breeding [1,2]. Due to the increased rate of parasite resistance, a combination therapy of many anthelmintic drugs has been used to achieve fast animal curing [2]. Q-DRENCH [3] is an oral suspension, containing four different active drugs with different pharmacological activities against GIT worms in sheep, commonly used in Australia and New Zeeland. The anti-parasitic drugs are Albendazole (ALB), Levamisole HCl (LEV), Abamectin (ABA), and Closantel (CLO); see Figure 1. Although co-administration of ALB and LEV causes the fast killing of the parasites if their concentrations exceed the minimum therapeutic effect in animal plasma, it cannot protect the animals from reinfection. Therefore, the addition of ABA and/or CLO greatly increases animals’ protection from recurrent infection [3]. Simultaneous analysis methods for drug mixtures are strongly desired in daily analysis in QC entities in pharmaceutical firms and both governmental and private drug investigation centers [4,5,6,7,8,9,10].

Critical evaluation of UV–spectrophotometric spectra of the four drugs indicates that ABA has the weakest UV absorbance due to the lack of many chromophoric groups if it is compared to the other three drugs [11,12].

General information about drugs’ chemistry and pharmacological activities can be summarized as follows. ALB’s [13,14] molecular formula is C_12_H_15_N_3_O_2_S, and its molecular weight is 265.33 g/mol. It is a derivative of benzimidazole, and it has strong anthelmintic activities against many parasites, i.e., cestodes and nematodes, via inhibition of the glucose uptake process by the worms. LEV’s [13,15] formula is C_11_H_12_N_2_S and its weight is 204.29 g/mol. LEV is killing ascariasis completely in humans. Moreover, it is also active against the hookworm parasite, while LEV has very poor efficiency against enterobiasis and trichuriasis. Furthermore, its racemic form, tetramisole, has less anthelmintic activity than LEV. ABA belongs to macrocyclic lactones 13, its molecular weight is 1732.1 g/mol, and its formula is C_95_H_142_O_28_. ABA exists in two forms, as a natural product, avermectin B1a (R = ethyl group), which composes approximately 80% or more, and avermectin B1b (R = methyl group), which is a semisynthetic compound and composes approximately 20%. ABA15 is commonly used for its pesticide effect for pests and parasitic worms because of its anthelmintic and insecticidal qualities. Finally, CLO’s [13,16] formula is C_22_H_13_Cl_2_I_2_N_2_NaO_2_, and its weight is 685.1 g/mol. CLO is a halogenated derivative with a salicylanilide base, with a powerful, long-acting anti-parasitic action. CLO is commonly used for the treatment of parasites in infected sheep. However, humans cannot use CLO because of its toxicity towards the CNS, i.e., it leads to ataxia and blindness in the case of overdose.

No simultaneous analytical method has been reported for the four anthelmintic drugs yet. However, a paper published recently, in 2019 [17], described the analysis of 40 anthelmintic drugs, including the investigated drugs, in water samples in Ireland using the LC-MS/MS technique after extraction with the usage of a divinylbenzene cartridge. The method was extremely sensitive as the detection level was in nanograms per liter. The method has also many drawbacks: it requires complex primary adsorption and desorption steps, and each anthelmintic drug has its specific analysis conditions, i.e., mobile phase composition, pH, collision energy, collision voltage, and ionization mode. In addition, the method could not be considered for the straightforward simultaneous analysis of the four veterinary drugs. Furthermore, many HPLC techniques have been recorded in the literature for the determination of the investigated drugs either alone [12,18,19,20,21,22,23,24,25] or in binary mixtures [26,27,28,29,30] or multi-drug mixtures [31]. However, none of these methods could be used for the direct quantitative analysis of the studied quaternary anthelmintic mixture in QC units. Developing one chromatographic condition for the quantitative analysis of complex drug mixtures is not an easy task in analytical chemistry laboratories, where many trials performed by expert analysts are needed, in addition to the scientific prediction of the chromatographic behaviors of drugs, which are usually related to their chemical structures. A UV detector is the most prevalent detector in HPLC methods; it was successfully used for the analysis of hepatoprotective drugs, vitamin E, and veterinary mixtures [7,10]. The greenness qualities of analytical techniques should be considered during their development and validation [32,33,34]. A HPLC stability study protocol [35,36,37] was followed for the analysis of the four drugs in an oral suspension in variable stress conditions (photo, thermal, acidic, basic, and oxidative conditions). The main goal of this research paper is to represent a direct and validated HPLC-UV method for their routine analysis in pure forms and pharmaceutical formulations. Furthermore, another objective is to check the stability of the four veterinary compounds in stressful degradation conditions. Consequently, we explore the optimal storage conditions for the Q-DRENCH formulation. Analysis of a quaternary mixture of drugs simultaneously is not an easy task for analysts. Till now, no direct HPLC method has been reported for the synchronized analysis of the four drugs that are present in the Q-DRENCH oral suspension, namely ABA, ALB, LEV, and CLO. Therefore, it is necessary to develop and validate a new, accurate, and simple chromatographic method for the regular QC analysis of the mentioned drugs without the interference of additives. Lastly, the AGREE tool is used for the evaluation of the method’s greenness.

## 2. Results and Discussion

### 2.1. Method Optimization

Different chromatographic items were considered in order to achieve acceptable separation. The first factor was the stationary phase, where C-18 gave reliable results if compared to the C-8 column. The second factor was the solvent type and ratio that were used in the organic mobile phase. Many solvents in different volumes were tried, e.g., methanol, ethanol, distilled water, ethyl acetate, and acetonitrile. The liquid system, with the ratio of 20:80 (water: acetonitrile, *v*/*v*), without adjusting the pH, showed acceptable resolution values but with tailed peaks. Triethylamine was added for ion pairing to improve the resolution and peak shapes, particularly for ALB and ABA. The control of the pH of the aqueous phase via glacial acetic acid to 3.5 resulted in a significant improvement for the outline of all peaks. Isocratic elution yielded very good results at a rate of 2 mL/min. Therefore, gradient elution was not attempted. Furthermore, the UV detector was successfully used for the detection of the drugs because of the observed absorbance in the region from 200 to 400 nm. Here, 210 nm was the optimal wavelength. The UV spectra for the four anthelmintic drugs are displayed in Supplementary Figure S1A–D. The use of 254 nm yielded poor sensitivity for most of the drugs. The novel RP-HPLC method separated the mixture in approximately 10 min.

**Figure 2:**
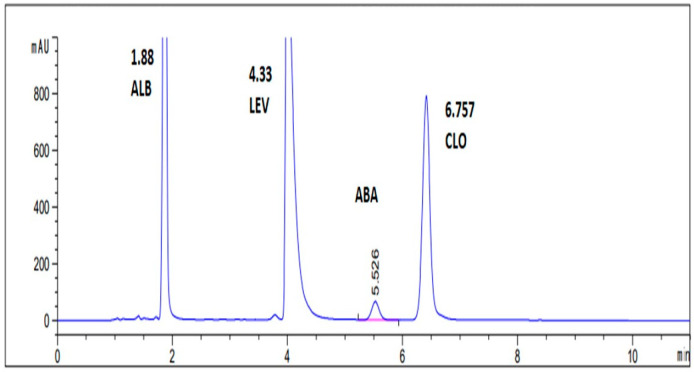
A typical chromatogram for the four separated pure anti-parasitic drugs ALB, LEV, ABA, and CLO at optimum chromatographic adjustments.

**Figure 3:**
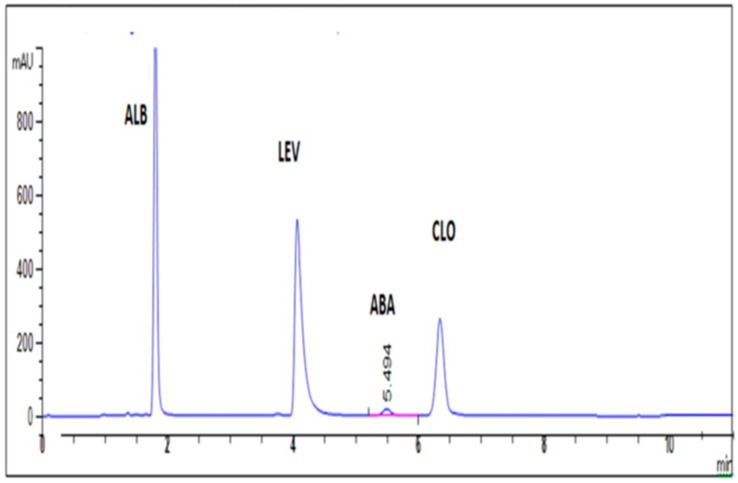
HPLC chromatogram of the separated mixture of ALB, LEV, ABA, and CLO in Q-DRENCH oral suspension.

Generally, the optimal HPLC performance was observed when using the C-18 stationary phase, Zorbax C18 (4.6 × 250 mm, 5 µm). The recommended liquid system was 1 mL of Triethylamine/L water, adjusting the pH to 3.5 by glacial acetic acid: acetonitrile solvent (20:80, *v*/*v*) at a flow rate of 2 mL per min in isocratic elution mode. The wavelength of 210 nm gave the maximum sensitivity at ambient temperature. Resolved symmetric peaks and satisfactory chromatograms are presented in Figure 2 and Figure 3.

### 2.2. Method Validation

The ICH protocol was followed during all steps of method validation [38].

#### 2.2.1. Estimation of Linearity Ranges

Linearity was assessed by measurement of five different concentrations of ALB, LEV, ABA, and CLO standards using the mentioned method in the Q-DRENCH oral suspension. Correlation coefficient values, which were ≥0.999, confirmed the satisfactory results of linearity for all four drugs.

Calibration curves were plotted, where the *Y*-axis represents the peak areas while the *X*-axis refers to the concentrations of the drugs. The calibration curves are illustrated in Supplementary Figure S2. In addition, the parameters of calibration equations, e.g., slope, intercept, and correlation coefficient, are displayed in Table 1.

#### 2.2.2. Accuracy

Accuracy was evaluated using three estimations over certain concentrations of ALB, LEV, ABA, and CLO within ranges of linearity. Accuracy was measured as the percentage recovery for the prepared concentrations. Samples were spiked by adding exact amounts of the standards to the placebo matrix containing all additives of the dosage form. The measurements were performed at different concentrations, which were 50%, 100%, and 150%. The listed results in Table 2 prove the accuracy of the new method. Moreover, the data in Table 2 confirm the absence of additive interference.

#### 2.2.3. Precision

The intra-day precision (*n* = 6) was calculated as the average of one concentration repeated six times on the same day while, the inter-day precision (*n* = 3) was estimated via the measurement of three replicates of a single sample on day 1, and then, on day 2, three repeats of fresh samples were examined. The same analyst performed both tests. The intra-day and day-to-day results summarized in Table 1 confirm the method’s precision. The full details of repeatability and intermediate precision are displayed in Supplementary Tables S1 and S2.

#### 2.2.4. Selectivity

The selectivity factor (α) and the resolution (Rs) data determined in Table 3 confirm the method’s selectivity, where all the values of α are higher than one and all the values of Rs are higher than 1.5. Furthermore, the well-separated peaks for the four drugs in Figure 3 and the results in Table 3 and Table 4 confirm the method’s selectivity, where no interference was detected from additives.

#### 2.2.5. Detection and Quantitation Limits (LOD and LOQ)

The results of LOD and LOQ are considered based on the equations shown in Table 1. Acceptable records for ALB, LEV, ABA, and CLO are illustrated in Table 1.

#### 2.2.6. System Suitability Parameters

The soundness of the presented chromatographic method was tested by recording the parameters of the system suitability that were generated automatically in chromatograms. Suitable results are enumerated in Table 3.

#### 2.2.7. Robustness

The method’s robustness was evaluated by recalculating the peak areas for ALB, LEV, ABA, and CLO after a slight change in mobile phase composition by the same analyst and within the same day. The calculated pooled RSDs were less than 3 for the four drugs, as illustrated in Table 4.

### 2.3. Confirmation of the Purity of the Four Drugs

The purity was estimated via comparison of the retention times for each tested drug in the veterinary suspension with its authentic standard (in a single chromatogram for each) and placebo solution as well. Identical retention times with at least one digit after the decimal were obtained for the four investigated drugs, as displayed in Table 5.

### 2.4. Application to the Q-DRENCH Oral Suspension for Sheep

In a veterinary oral suspension, many excipients are present, e.g., methyl paraben and propyl paraben. The two preservatives did not appear at the selected wavelength (210 nm) using the C-18 stationary phase. Upon using a 210 nm wavelength and C-18 column, only the four peaks for the drugs were present in the chromatogram, as displayed in Figure 3. Moreover, the data in Table 5 emphasize that the mentioned additives did not interfere with the main four peaks for the drugs. Therefore, this wavelength is selective for the four drugs only under optimal separation parameters, where none of the additives appeared in the Q-DRENCH chromatogram. Moreover, the concentrations of additives are very low and can be sensed at 210 nm.

The results for the quantitate analysis of ALB, LEV, CLO, and ABA in the Q-DRENCH suspension test product are recorded in Table 5, where the concentrations of ALB, LEV, CLO, and ABA are 62.5, 100.0, 93.8, and 100.0 µg/mL, respectively, in the Q-DRENCH suspension. Furthermore, reasonable recoveries were achieved for the four drugs using the standard addition technique in the Q-DRENCH formulation, as illustrated in Supplementary Table S3.

### 2.5. Results for the Forced Degradation Study

As illustrated in the chromatograms in Supplementary Figure S1, the peaks of the four drugs were well separated from other peaks for the degradation products that resulted from the different treatment conditions. The main peak areas for the four drugs were automatically recorded and the percentages of degradation were exactly calculated, as represented in Table 6. For ABA, the highest degradation was detected in the acidic medium (30.45%), while the lowest degradation was detected in the alkaline medium (7.43%). For ALB, the highest degradation was detected in the acidic medium (29.64%) while the lowest degradation was perceived in the H_2_O_2_ medium (1.45%). Moreover, for LEV, the highest degradation was detected in the photodegradation conditions (56.35%), while the lowest degradation was identified in the H_2_O_2_ medium (12.96%). Finally, for CLO, the highest degradation was detected in the basic medium (25.73%), while the lowest degradation was noticed in the acidic medium (14.10%).

### 2.6. Recommendations Based on the Outcomes of Stability Studies

The oral suspension should be stored away from direct light (especially for LEV), heat, oxidative, acidic (especially for ABA and ALB), and basic (especially for CLO) conditions.

### 2.7. Eco-Friendly Nature Estimation for the New HPLC Method

**Figure 4:**
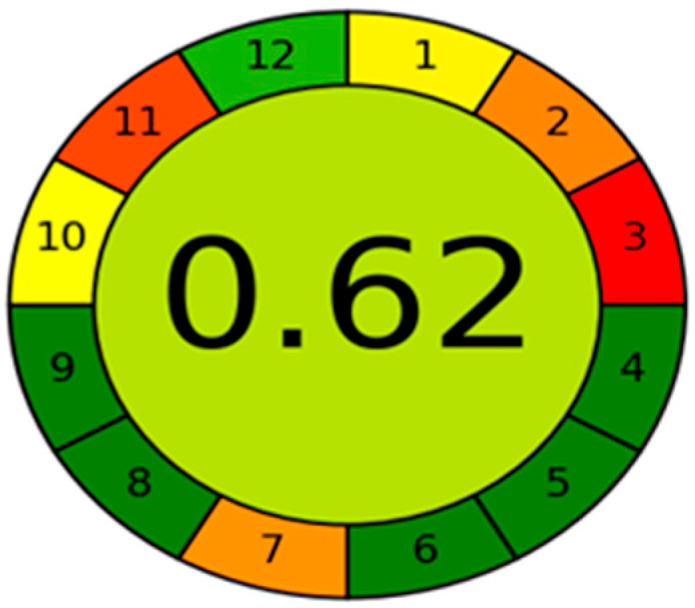
AGREE approach for estimation of method greenness for resolving a mixture of ALB, LEV, ABA, and CLO.

Among greenness tools, the AGREE approach was selected because of its simplicity, automation, and integration [32,39]. The total greenness score of the novel RP-HPLC method was 0.62, with a relatively faint green color in the middle of the pictogram that refers to the acceptable greenness quality of the method, as demonstrated in Figure 4. The use of acetonitrile was necessary to achieve the required separation, which is not preferred in terms of method greenness. The full data for the AGREE approach are displayed in Supplementary File S1. The main two subdivisions with the lowest scores in the pictogram are subdivision 3 and subdivision 11. Subdivision 3 refers to off-line sampling, while subdivision 11 denotes the used toxic reagents. The analysis of the four drugs within ten minutes only confirms the suggestion of method greenness as described in the fully green subdivision 8, where multi-samples are determined in a single run and approximately 24 samples could be analyzed in 1 h.

### 2.8. Future Research and Limitations of the Study

Because the first goal of this work was to develop a simple RP-HPLC method in pharmaceuticals for regular QC use, the proposed RP-HPLC method was not tested with biological fluids from animals. Moreover, the pharmacokinetic profiles of the four active ingredients need to be re-estimated concurrently in real animal samples. Additionally, the purity of each peak could be further checked using a mass spectrophotometric detector.

## 3. Materials and Methods

### 3.1. Apparatus

For quantification purposes, an Agilent 1200 series HPLC instrument (Agilent Technologies, Santa Clara, CA, USA) was used, which consisted of an online degasser (G1322A), a quaternary pump (G1312A), an auto-sampler (G1367C), a column temperature regulator (G1316A), and a UV detector (G1315B). Chromatographic experiments were carried out on an Agilent C18 reversed-phase column (4.6 × 250 mm, 5 μm). A sonicator instrument was used, produced by Memmert Co. (Schwabach, Germany).

### 3.2. Materials

#### 3.2.1. Chemicals and Pure Drug Samples

Glacial acetic acid was obtained from Alnasr Co. (Cairo, Egypt). Tri-Ethylamine and methanol were obtained from Sigma Aldrich (Steinheim, Germany). Purified water was prepared in the lab via a double distillation process and filtered by a 0.45 μm membrane filter. The degassing process was accomplished and the liquid movable phase was cleaned via a 0.45 μm Millipore filter (Sartorius, Germany).

ALB, LEV, ABA, and CLO pure samples were imported from Sandoz UK as gifts. The purity for each drug was 99.88%, 99.58%, 99.98%, and 100.21%, respectively, according to company certificates.

#### 3.2.2. Pharmaceutical Formulations

Q-DRENCH oral suspension for sheep contains 2.5% (25 g/L) ALB, 4% (40.0 g/L) LEV, 0.1% (1.0 g/L) ABA, and 3.75% (37.5 g/L) CLO; it was manufactured by JUROX Animal Health Company (Rutherford, Australia).

### 3.3. Preparation of Pure Standard’s Working and Stock Organic Solutions

All solutions were prepared in the mobile phase solution for each drug as follows:

Stock solutions (A1): 200 mg of LEV, 187.5 mg of CLO, and 125 mg of ALB were separately transferred to 100 mL volumetric flask; then, seventy mL of mobile phase was added and sonicated for 20 min. Finally, the volume was filled with the liquid mobile phase.

While stock solution (A2) for ABA was prepared as follows: 25 mg of ABA was moved to a 250 mL volumetric flask; then, 200 mL of the liquid mobile system was added and sonicated for 20 min. The final size was completed with the liquid mobile system.

Working solutions (B): 5 mL from solution (A1) was moved to a 100 mL volumetric flask and diluted to the final capacity by the organic mobile phase.

### 3.4. Preparation of Q-DRENCH Oral Suspension Solution

Regarding LEV, CLO, and ALB, 5 mL of Q-DRENCH oral suspension (equiv. to 200 mg LEV, 187.5 mg CLO, 100 mg ALB for each) was carefully moved to a 100 mL volumetric flask. After this, 70 mL of the liquid mobile phase was added and sonicated for 20 min. The flask was filled to the end with the liquid mobile phase. After this, working solutions (B) were prepared by dilution with liquid mobile phase, as mentioned in Section 2.3.

Regarding ABA, a new working solution was prepared as follows: 10 mL of Q-DRENCH oral suspension was carefully moved to a 100 mL volumetric flask. After this, 70 mL of the liquid mobile phase was added and sonicated for 20 min. Lastly, the flask was filled to the end with the liquid movable system.

### 3.5. General Chromatographic Procedures

Many trials were performed to optimize the chromatographic conditions. The best chromatogram was attained by the use of the following parameters, as illustrated in Table 7. Generally, three drugs, namely ALB, LEV, and CLO, were estimated in one run, while ABA was determined in a separate run under the same operating conditions because of the low concentration of ABA in the Q-DRENCH oral suspension compared to the other three drugs.

### 3.6. Processes for Stability Studies for the Four Drugs

The HPLC stability study protocol [35,36,37] was followed for the analysis of the four drugs in the oral suspension under variable stress conditions (photo, thermal, acidic, basic, and oxidative conditions). The analyzed solutions containing the investigated drugs were prepared and compared to blank chromatograms. Placebo solutions without treatment were prepared by adding all inactive ingredients without the addition of the drugs. Then, stress degradation settings were applied as in the tested solutions. The stress degradation settings are recorded in Table 7 and were applied simultaneously for both placebo and test solutions as well.

### 3.7. Assessment of Eco-Friendly Qualities of the Novel HPLC Method via AGREE Tool

The analytical method should be evaluated in terms of its greenness quality for the sake of the environment and chemists as well. The AGREE software is an automated, reliable, qualitative, and quantitative tool [32,39]. The twelve principles of green analytical chemistry are the main components of the AGREE approach [39]. The data of the new HPLC method were used to generate both the full report and the three-colored pictogram.

## 4. Conclusions

A reliable, simple, precise, and stability-indicating RP-HPLC method for the quantitative analysis of ALB, LEV, ABA, and CLO has been proposed. The novel method could be used for routine QC analysis of the four drugs in pure powders and oral suspension without interfering excipients. The AGREE pictogram for the RP-HPLC method suggests the dependability of the novel method in terms of the greenness point of view. The validation parameters were evaluated, as recommended by ICH guidelines. The oral suspension Q-DRENCH should be stored away from direct light, heat, oxidative, acidic, and basic conditions.

## Figures and Tables

**Table 1:** Validation results of the recommended HPLC method for analysis of ALB, LEV ABA, and CLO.

Parameters	ALB	LEV	ABA	CLO
Range in (µg/mL)	15.15–93.75	25–150	30–150	11.7–140.63
Slope (mAU*s mL/µg)	32.252	23.239	4.174	18.312
Intercept (mAU*s)	−9.008	−26.128	2.028	4.568
R2	0.99984	0.99980	0.99997	0.99990
Accuracy (mean ± SD)	101.06 ± 0.489	101.15 ± 0.579	99.61 ± 0.588	100.58 ± 0.723
Precision (%RSD) Repeatability * RSD ≤ 2% Day to day precision **	0.47	0.33	0.35	1.25
Pooled RSD ≤ 3%	1.47	1.62	0.29	2.03
LOD in (µg/mL) ***	1.669	2.989	1.167	1.991
LOQ in (µg/mL) ***	5.056	9.057	3.537	6.033

* The intra-day precision (*n* = 6), average of one concentration repeated six times in the same day. ** The inter-day precision (*n* = 3), 3 replicates of a single sample were implemented on the first day, and then, on a second day, 3 replicates of freshly prepared samples were analyzed. The same analyst performed both tests. *** Limits for detection and quantitation were calculated via LOD = (SD of the response/slope) × 3; LOQ = (SD of the response/slope) × 10.

**Table 2:** Accuracy results obtained by the proposed HPLC method after standard addition of 50% to 150% including 100% of the target concentration.

**Drug Name**	**ALB**		**LEV**	
**Conc (%)**	Average Peak Area (mAU*s)	**Recovery (%)**	Average Peak Area (mAU*s)	**Recovery (%)**
50%	1003.0	101.14%	1134.3	100.49%
100%	2015.3	101.61%	2300.5	101.90%
150%	2987.6	100.42%	3422.6	101.07%
**Drug name**	**ABA**		**CLO**	
Conc (%)	Average peak area (mAU*s)	Recovery (%)	Average peak area (mAU*s)	Recovery (%)
50%	207.2	100.34%	869.0	101.49%
100%	408.5	98.90%	1721.6	100.53%
150%	617.0	99.58%	2561.7	99.72%

**Table 3:** System suitability parameters of RP-HPLC method for quantitative analysis of ALB, LEV, CLO, and ABA.

Parameters	Values				Reference Value
	ALB	LEV	CLO	ABA *	
R_t_ (min)	1.88	4.33	6.76	5.53	–
Resolution (R)	16.2		11.10		R > 1.5
Selectivity factor (α)	2.27		1.58		>1
Symmetry factor “Tailing factor” (T)	1.25	1.61	1.08	1.05	≈1
Column efficiency (N)	7228	6962	12,443	8501	Increase with efficiency of the separation
HETP ^b^	0.0035	0.0036	0.002	0.0029	The lower the value, the higher the efficiency of the analytical column

HETP ^b^ = height equal to theoretical plate, (cm/plate). * Data for ABA were taken as recorded automatically from chromatogram after a separate run for ABA.

**Table 4:** Results of method robustness for analysis of ALB, LEV, CLO, and ABA (slight change in mobile phase composition).

Replicate	ALB		LEV		CLO		ABA	
	Condition 1	Condition 2	Condition 1	Condition 2	Condition 1	Condition 2	Condition 1	Condition 2
1	1984.16	2018.49	2286.96	2308.80	1696.93	1754.22	426.648	417.386
2	2003.96	1998.72	2286.48	2313.65	1703.78	1706.78	425.807	417.432
3	2003.50	1990.95	2275.82	2301.01	1695.26	1710.67	428.765	416.438
Pooled mean Peak areas (mAU*s)	2000.0		2295.5		1711.3		422.1	
Pooled SD	11.9		14.7		21.8		5.6	
Pooled RSD Accepted criteria ≤ 3%	0.59		0.64		1.28		1.32	

Condition 1: optimum conditions. Condition 2: 1 mL of Triethylamine/L water, adjusting pH to 3.5 by glacial acetic acid: acetonitrile (25:75), *v*/*v*.

**Table 5:** Results of method specificity/selectivity for the analysis of ALB, LEV, CLO, and ABA in Q-DRENCH oral suspension by the new HPLC method.

	**ALB**			**LEV**		
Test Name	Conc (µg/mL)	Peak RT (min)	Peak Area (mAU*s)	Conc (µg/mL)	Peak RT (min)	Peak Area (mAU*s)
Standard	62.5	1.894	2004.78	100.0	4.311	2307.77
Q-DRENCH suspension test	62.5	1.892	1988.94	100.0	4.304	2255.12
Placebo	0	No peak observed	No peak observed	0	No peak observed	No peak observed
	**CLO**			**ABA**		
Standard	93.8	6.757	1695.07	100.0	5.494	414.11
Q-DRENCH suspension test	93.8	6.765	1684.66	100.0	5.498	409.28
Placebo	0	No peak observed	No peak observed	0	No peak observed	No peak observed

**Table 6:** The percentage of recovery and degradation of the four drugs after forced degradation parameters.

Degradation Type	Conditions	ABA		ALB		LEV		CLO	
		Assay%	Degradation%	Assay%	Degradation%	Assay%	Degradation%	Assay%	Degradation%
Light	Light (48 h)/UV (12 h)	74.50	25.50	79.04	20.96	43.65	56.35	85.72	14.28
Heat	80 °C (8 h.)	82.92	17.08	75.45	24.55	65.32	34.68	80.59	19.41
Acid	1N HCl/80 °C (1 h)	69.55	30.45	70.36	29.64	65.19	34.81	85.90	14.10
Base	1N NaOH/80 °C (1 h)	92.57	7.43	75.75	24.25	76.07	23.93	74.63	25.37
H_2_O_2_	0.5% H_2_O_2_/80 °C (1 h)	88.86	11.14	98.55	1.45	87.04	12.96	81.24	18.76

**Table 7:** Chromatographic conditions for separation of ALB, LEV, ABA, and CLO mixture in Q-DRENCH oral suspension.

Instrument:	Agilent HP1200 or equivalent
System Type:	Reverse Phase
Column Type:	Zorbax C18
Length and Diameter:	4.6 × 250 mm, 5 µm
Conditioning	With mobile phase for 45 min prior to operating the injection sequence
Column Temperature:	Ambient
Injection Type and Volume:	Auto Sampler, 10 µm
Detector Type:	UV Detector
Wavelength:	210 nm
Mobile Phase Composition:	1 mL of Triethylamine/L water, adjusting pH to 3.5 by glacial acetic acid: acetonitrile (20:80), v/v
Flow Rate:	2.0 mL/min

## Data Availability

Not applicable.

## Supplementary Materials

Figures S1 and S2: Figures for UV spectra for the four drugs, calibration curves, and stability studies chromatograms; Tables S1–S3: Ruggedness results and results of standard addition technique in the Q-DRENCH formulation; File S1: Analytical greenness report sheet.
