# Supplementary material for: Quantitative Analysis of Abamectin, Albendazole, Levamisole HCl and Closantel in Q-DRENCH Oral Suspension Using a Stability-Indicating HPLC-DAD Method
Source: Molecules. 2022 Jan 24;27(3):764. doi: 10.3390/molecules27030764 (PMC8839283; doi:10.3390/molecules27030764)
Supplement: Supplementary file 1 [file molecules-27-00764-s001.zip › Supplementary Tables 17-1-2022.pdf]

Table S1: G- Ruggedness results:

Day to day results

Replicate	Albendazole		Levamisole HCl		Abamectin		Closantel		Acceptance criteria
	Day 1	Day 2	Day 1	Day 2	Day 1	Day 2	Day 1	Day 2	
**1**	2128.02	2181.64	2430.02	2501.82	433.787	435.245	1813.52	1908.48	
**2**	2129.07	2186.61	2435.22	2501.21	434.770	432.830	1815.23	1862.91	
**3**	2132.25	2193.71	2435.62	2515.18	436.405	433.937	1823.48	1864.17	
**Pooled Mean**	**2158.5**		**2469.8**		**434.5**		**1848.0**		
**Pooled SD**	**31.8**		**40.0**		**1.3**		**37.4**		
**Pooled RSD**	**1.47**		**1.62**		**0.29**		**2.03**		**≤ 3%**

Comment: Method is rugged as the pooled RSD is not more than 3%.

Table S2: Analyst to analyst results

Replicate#	Albendazole		Levamisole HCl		Abamecitin		Closantel		Acceptance criteria
	Analyst 1	Analyst 2	Analyst 1	Analyst 2	Analyst 1	Analyst 2	Analyst 1	Analyst 2	
**1**	2058.7	2010.2	2352.2	2290.7	431.082	418.296	1756.7	1706.0	
**2**	2053.5	2006.6	2352.8	2279.6	431.751	416.683	1751.3	1703.7	
**3**	2048.1	2002.9	2342.2	2271.7	432.738	417.007	1755.1	1703.2	
**Pooled Mean**	**2030.0**		**2314.9**		**424.6**		**1729.3**		
**Pooled SD**	**26.0**		**38.1**		**8.0**		**27.5**		
**Pooled RSD**	**1.28**		**1.65**		**1.88**		**1.59**		**≤ 3%**

Comment: Method is rugged as the pooled RSD is not more than 3

Table S3: Results of standard addition technique in the Q-DRENCH formulation

ALB		LEV		CLO		ABA	
Conc.	recovery	Conc.	recovery	Conc.	recovery	Conc.	recovery
31.25	97.55	50	98.15	46.88	97	50	98.46
46.88	97.08	75	98.67	70.31	97.27	75	97.57
62.5	98.46	100	99.04	93.75	98.34	100	97.39
78.13	97.66	125	98.92	117.19	97.16	125	98.07
93.75	98.09	150	97.97	140.63	98.18	150	97.16
