# Supplementary material for: Quantitative Analysis of Abamectin, Albendazole, Levamisole HCl and Closantel in Q-DRENCH Oral Suspension Using a Stability-Indicating HPLC-DAD Method
Source: Molecules. 2022 Jan 24;27(3):764. doi: 10.3390/molecules27030764 (PMC8839283; doi:10.3390/molecules27030764)
Supplement: Supplementary file 1 [file molecules-27-00764-s001.zip › supplemtry file 1.pdf]

Analytical Greenness report sheet

22/09/2021 13:50:20

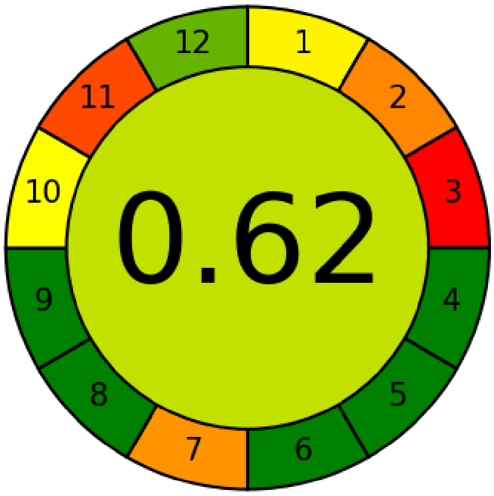

Criteria	Score	Weight
1. Direct analytical techniques should be applied to avoid sample treatment.	0.48	2
2. Minimal sample size and minimal number of samples are goals.	0.27	2
3. If possible, measurements should be performed in situ.	0.0	2
4. Integration of analytical processes and operations saves energy and reduces the use of reagents.	1.0	2
5. Automated and miniaturized methods should be selected.	1.0	2
6. Derivatization should be avoided.	1.0	2
7. Generation of a large volume of analytical waste should be avoided, and proper management of analytical waste should be provided.	0.29	2
8. Multi-analyte or multi-parameter methods are preferred versus methods using one analyte at a time.	1.0	2
9. The use of energy should be minimized.	1.0	2
10. Reagents obtained from renewable sources should be preferred.	0.5	2
11. Toxic reagents should be eliminated or replaced.	0.14	2
12. Operator's safety should be increased.	0.8	2
