# Supplementary material for: Quantitative Analysis of Abamectin, Albendazole, Levamisole HCl and Closantel in Q-DRENCH Oral Suspension Using a Stability-Indicating HPLC-DAD Method
Source: Molecules. 2022 Jan 24;27(3):764. doi: 10.3390/molecules27030764 (PMC8839283; doi:10.3390/molecules27030764)
Supplement: Supplementary file 1 [file molecules-27-00764-s001.zip › supplymentry figures 17-1-2022.pdf]

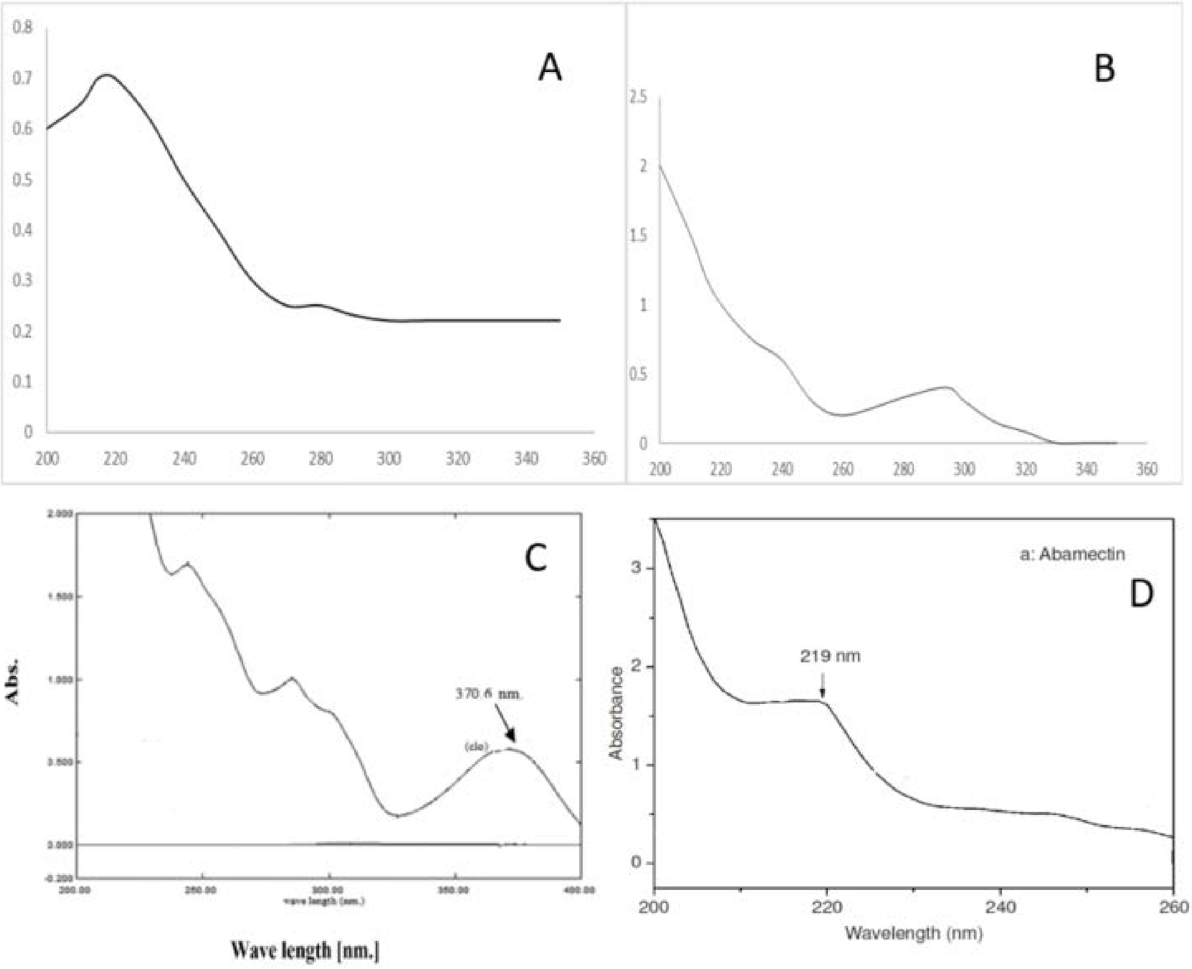

Figure S1: UV spectrum of levamisole in water(A); UV spectrum of albendazole in water (B); UV spectrum for Closantel Sodium in 50 % aqueous acetonitrile(C); UV spectrum for Abamectin in water(D).

Albendazole Calibration curve

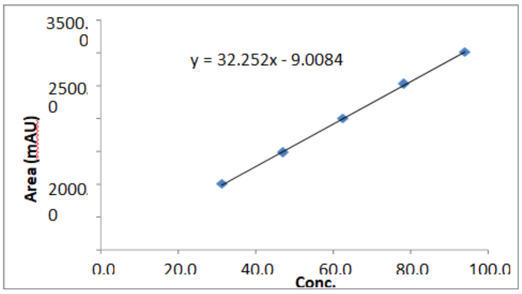

Levamisole HCl Calibration curve

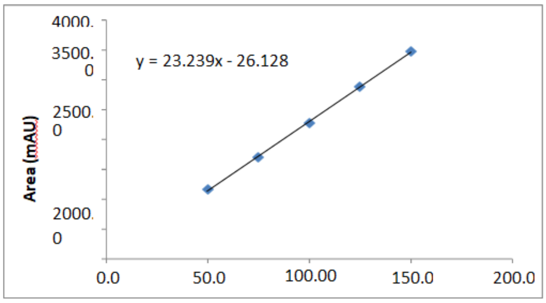

Abamectin Calibration curve

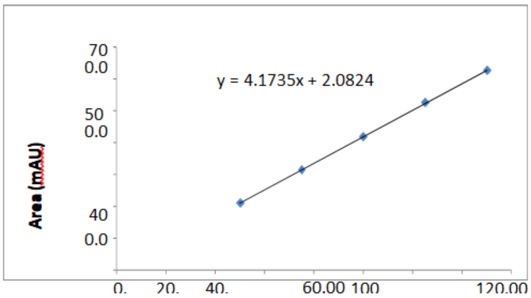

Closantel Calibration curve

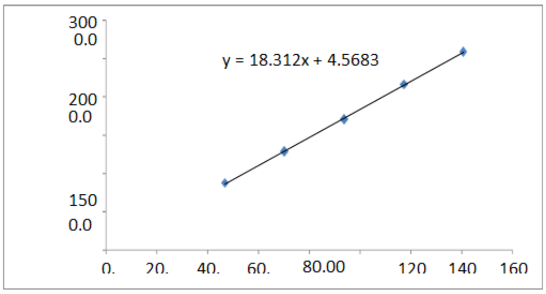

Figure S2: Calibration curves for the analysis of the four drugs using the new HPLC method

Sample Name: leva_albenda_clos pure standards.

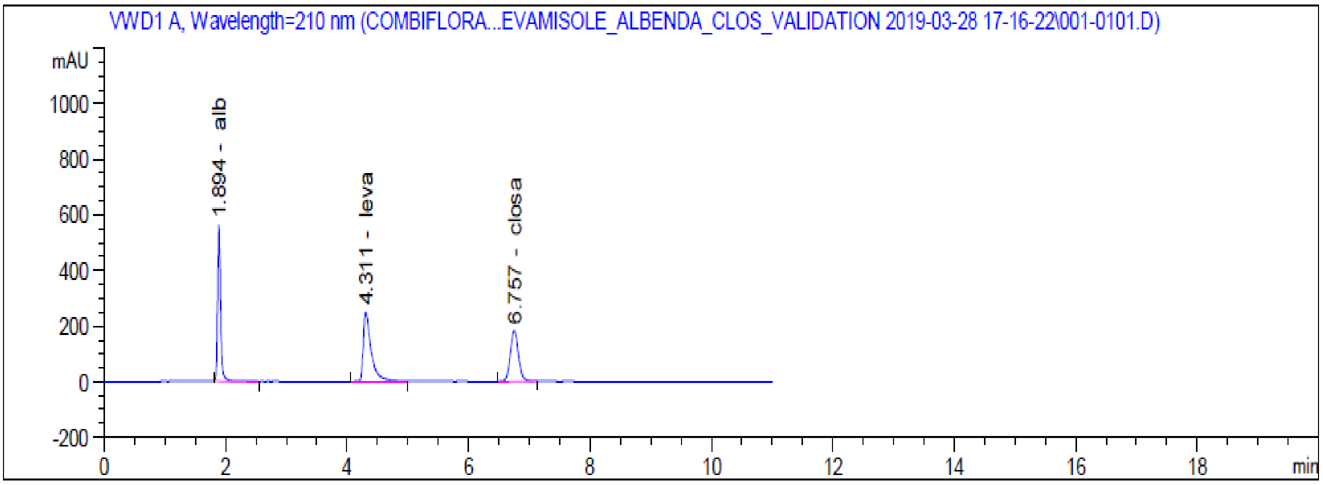

Sample Name: leva_albenda_clos dosage form chromatogram

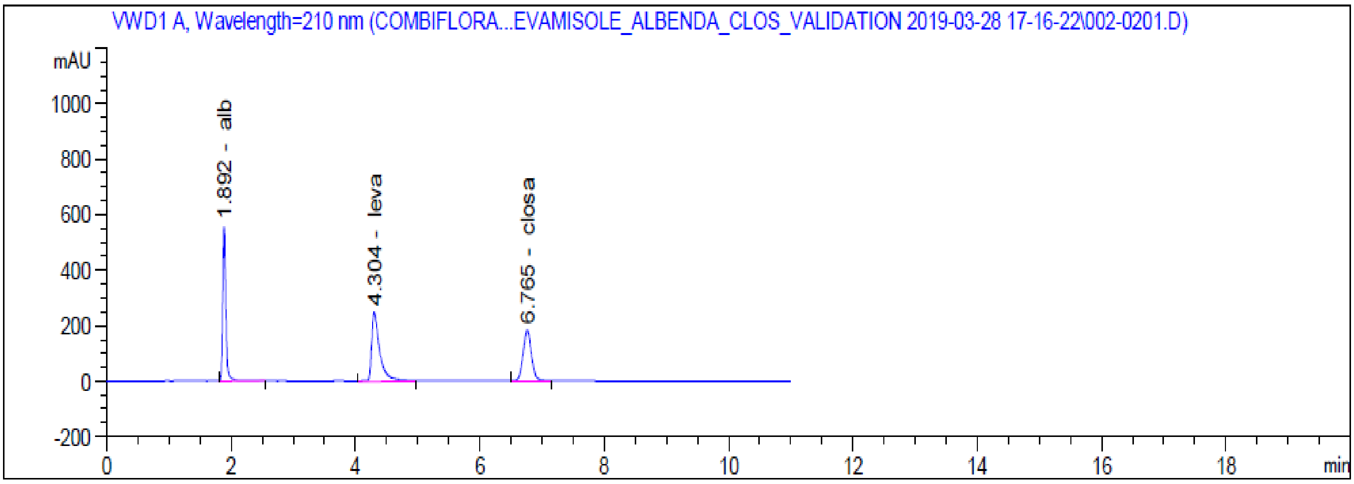

Sample Name: leva_albenda_clos Standard placebo

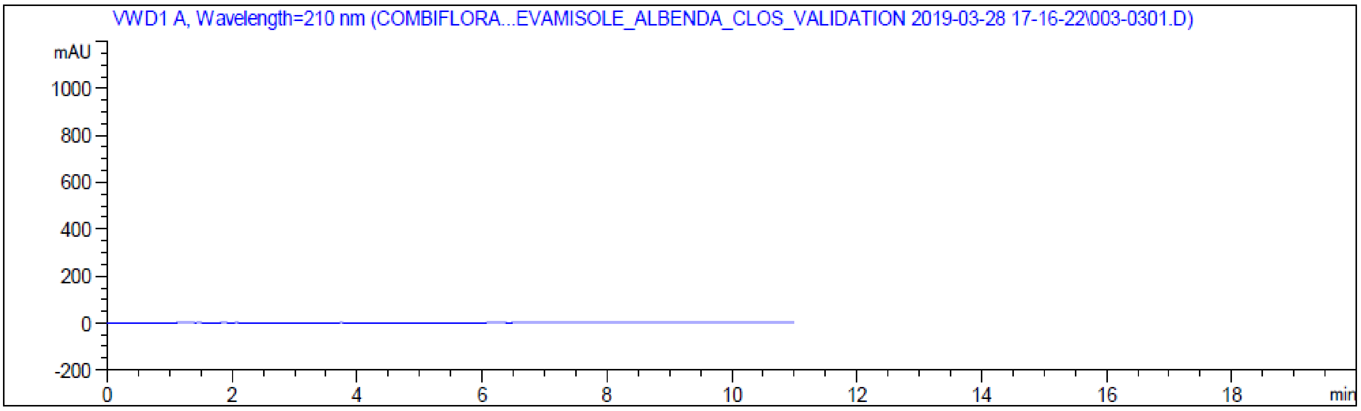

Sample Name: leva_albenda_clos standard placebo

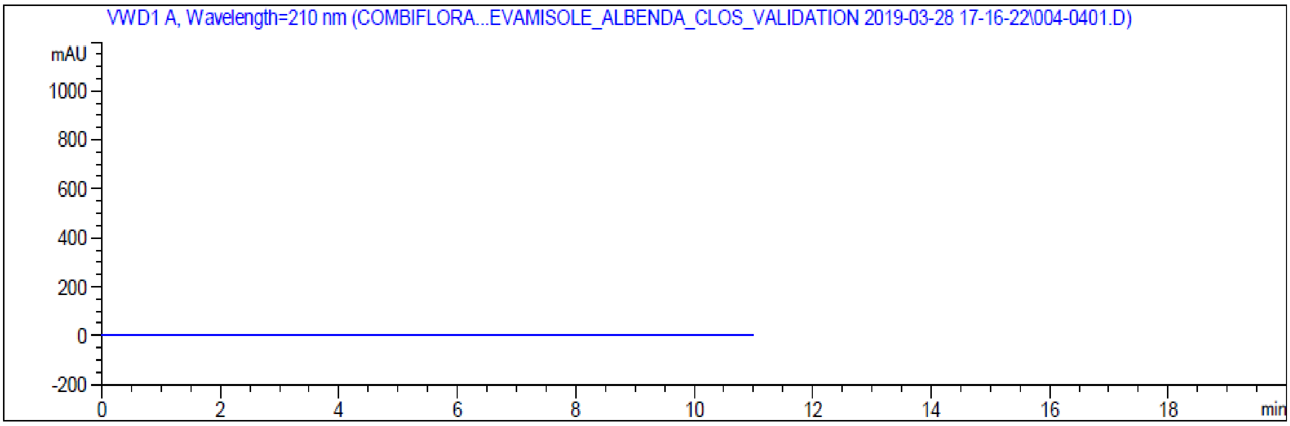

Sample Name: leva_albenda_clos photo degradation chromatogram

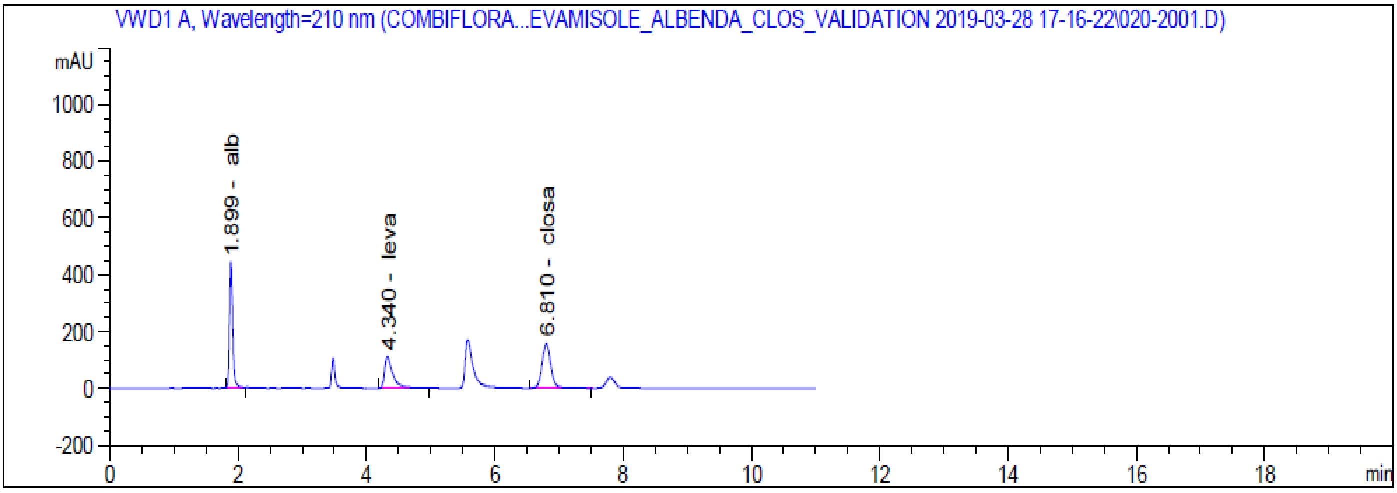

Data File C:\CHEM32\...E\LEVAMISOLE_ALBENDA_CLOS_VALIDATION 2019-03-28 17-16-22\021-2101.D

Sample Name: leva_albenda_clos heat degradation chromatogram

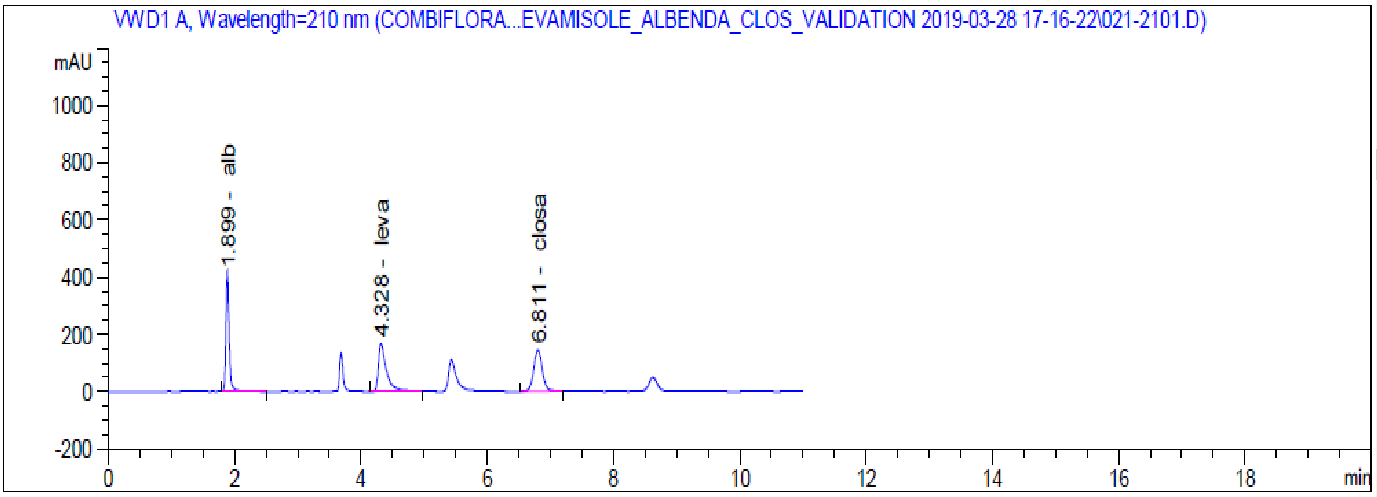

Sample Name: leva_albenda_clos acid degradation chromatogram

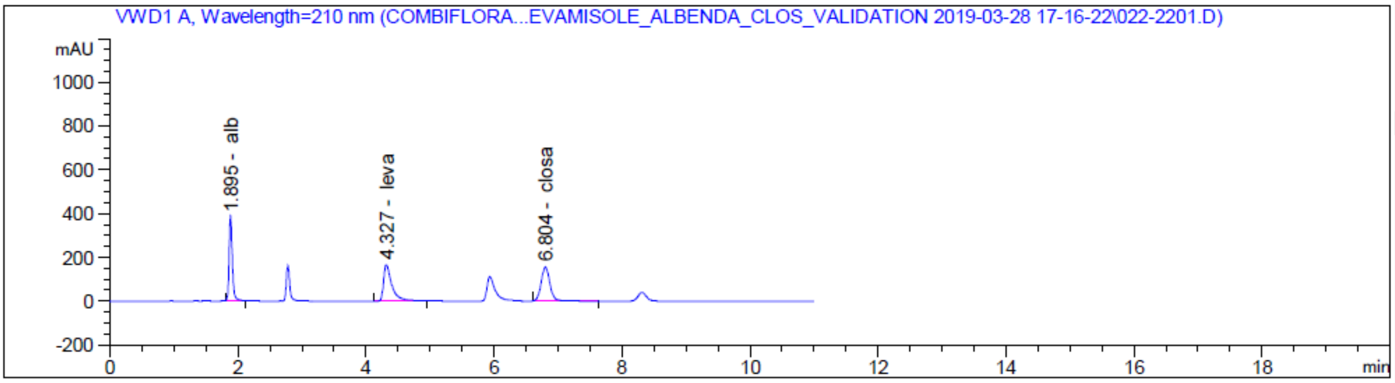

Data File C:\CHEM32\...E\LEVAMISOLE_ALBENDA_CLOS_VALIDATION 2019-03-28 17-16-22\023-2301.D

Sample Name: leva_albenda_clos base degradation chromatogram

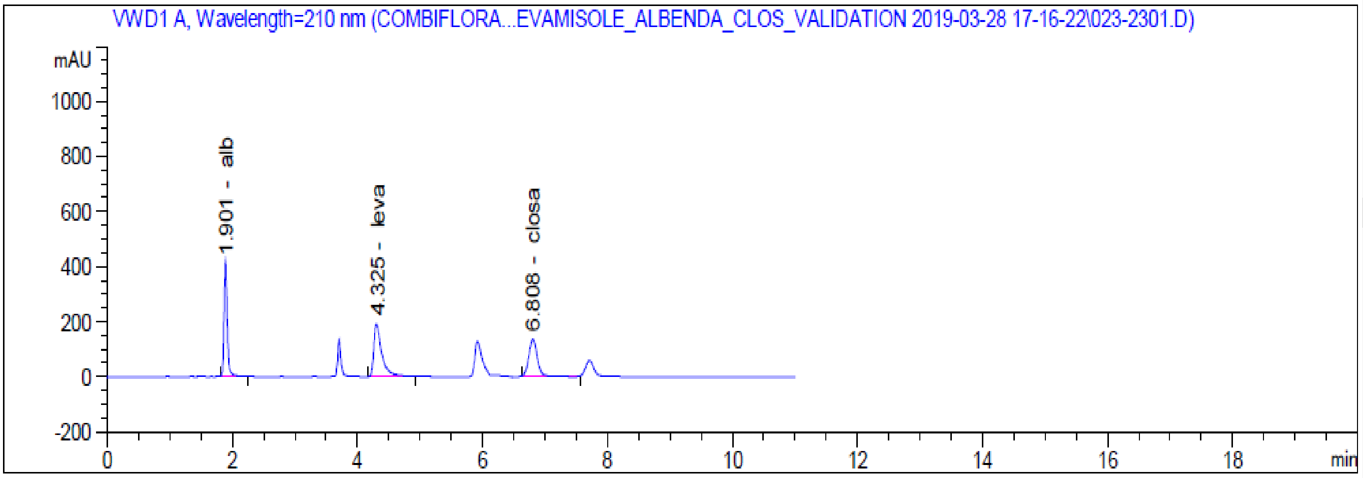

Sample Name: leva_albenda_clos oxidative degradation chromatogram

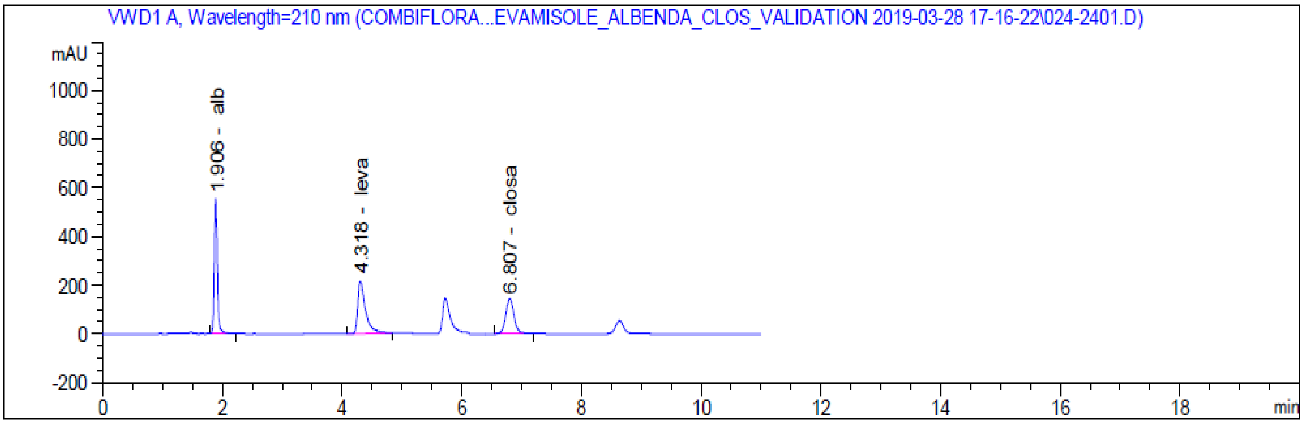

Sample Name: abamectin tested dosage form (placebo)

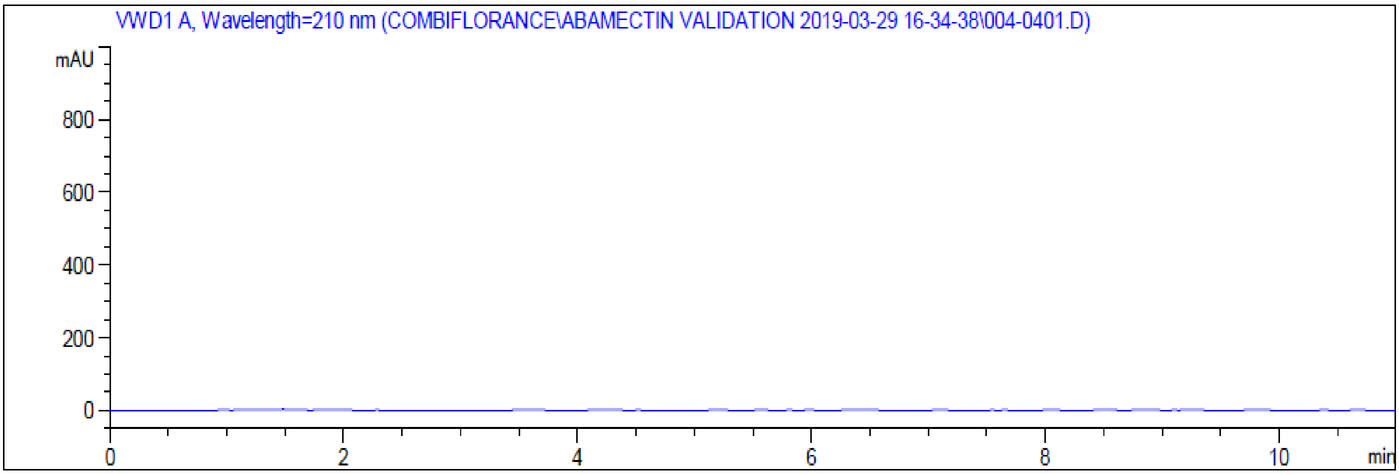

Sample Name: abamectin photo degradation chromatogram

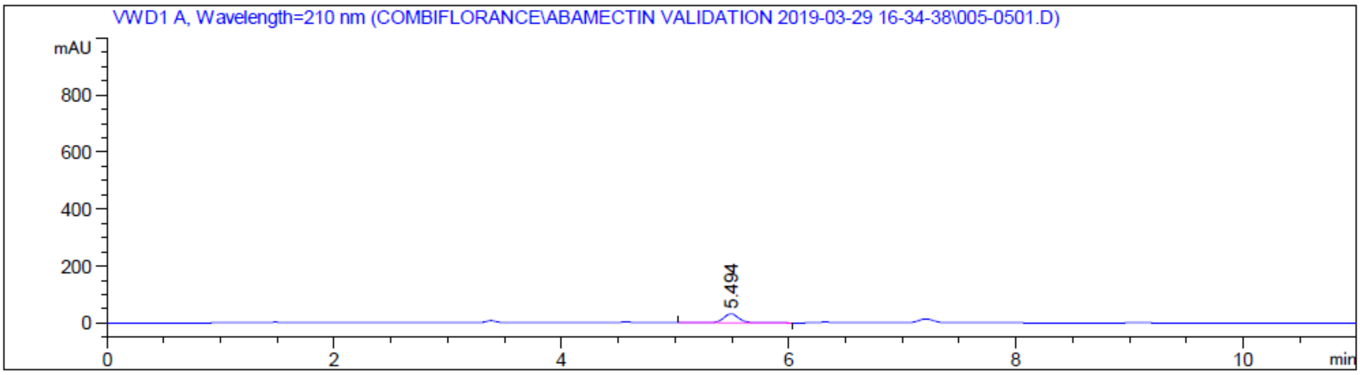

Sample Name: abamectin heat degradation chromatogram

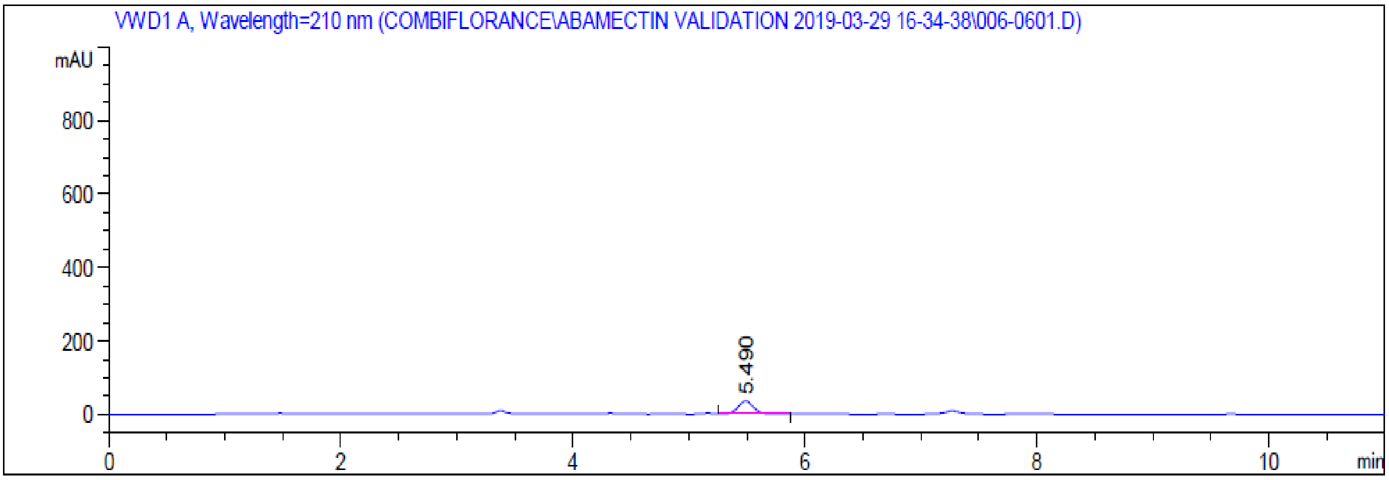

Sample Name: abamectin acid degradation chromatogram

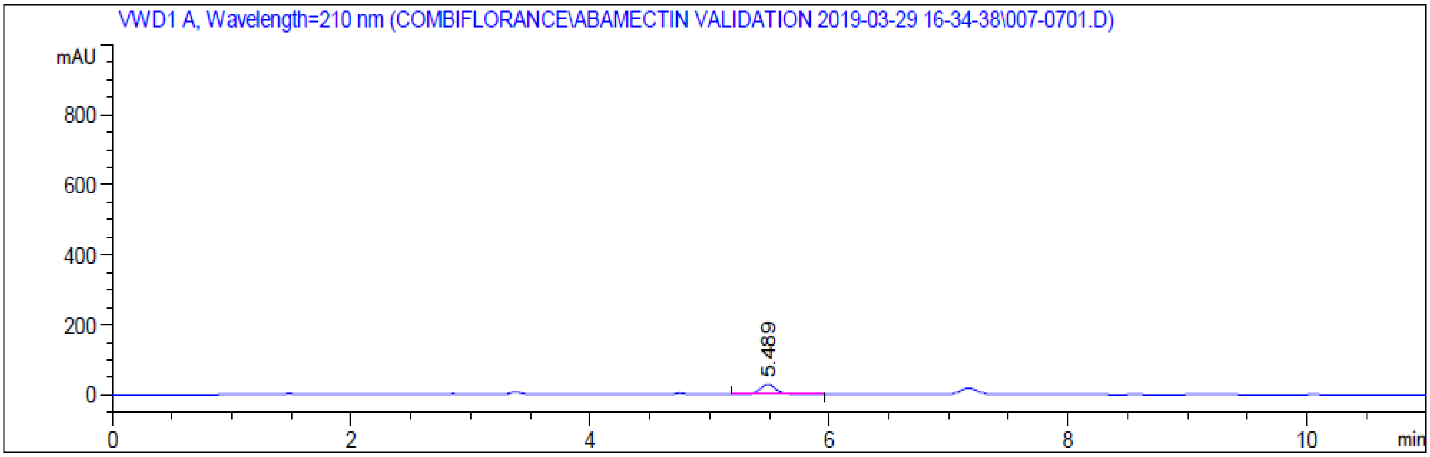

Sample Name: abamectin base degradation chromatogram

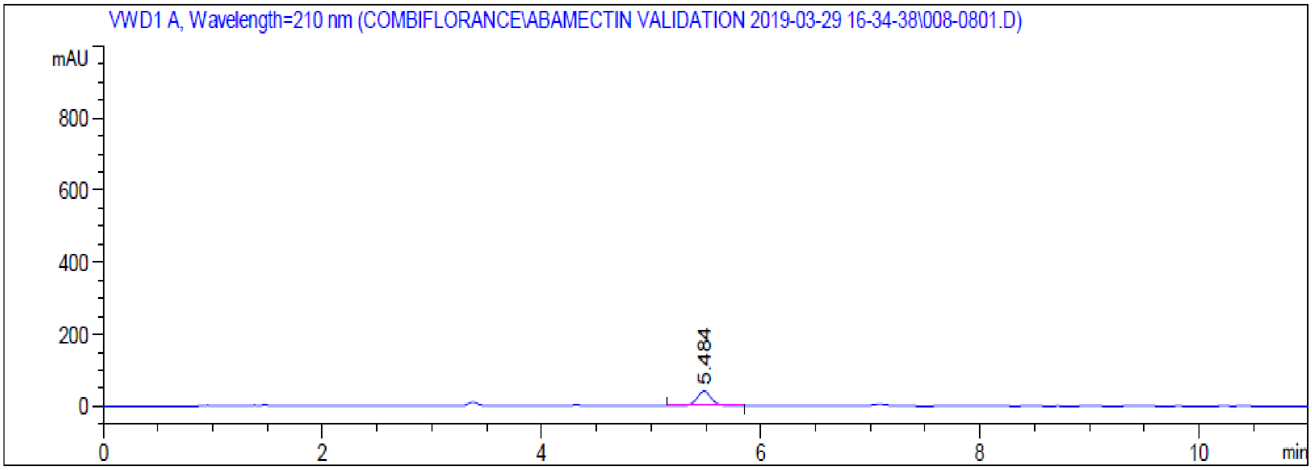

---------- --------- -------------- ----------------------- ------------ ---------------- ---------- ------------ ---------------

Sample Name: abamectin oxidative degradation chromatogram

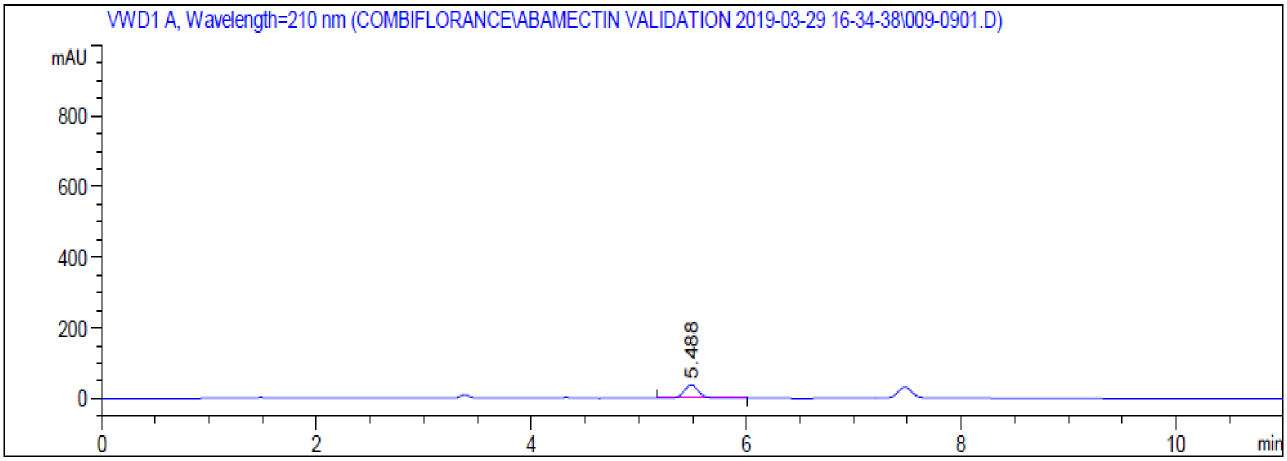

---------- --------- -------------- ---------------------- ------------ ---------------- ---------- ------------ --------------
